# Study of the external female genitalia of 14 *Rhodnius* species (Hemiptera, Reduviidae, Triatominae) using scanning electron microscopy

**DOI:** 10.1186/1756-3305-7-17

**Published:** 2014-01-09

**Authors:** João Aristeu da Rosa, Vagner José Mendonça, Sueli Gardim, Danila Blanco de Carvalho, Jader de Oliveira, Juliana Damieli Nascimento, Heloisa Pinotti, Mara Cristina Pinto, Mario Cilense, Cleber Galvão, José Maria Soares Barata

**Affiliations:** 1 Departamento de Ciências Biológicas, Faculdade de Ciências Farmacêuticas, Universidade Estadual Paulista Araraquara, Rodovia Araraquara-Jaú km 1, 14 801-902, Araraquara, SP, Brasil; 2 Departamento de Físico-Química, Instituto de Química, Universidade Estadual Paulista, Araraquara, Av. Prof. Francisco Degni, 55, 14 800-060 Araraquara, SP, Brasil; 3 Departamento de Biologia Animal, Instituto de Biologia, Universidade Estadual de, Campinas, Campinas, SP, Brasil; 4 Centro Universitário de Araraquara - UNIARA, Araraquara, SP, Brasil; 5 Departamento de Entomologia, Instituto Oswaldo Cruz, Laboratório Nacional e Internacional de Referência em Taxonomia de Triatomíneos, Rio de Janeiro, RJ, Brasil; 6 Departamento de Epidemiologia, Faculdade de Saúde Pública, São Paulo, SP, Brasil

**Keywords:** Triatominae, *Rhodnius*, External female genitalia, Scanning electron microscopy

## Abstract

**Background:**

Among the vectors of Chagas disease (Hemiptera: Reduviidae:Triatominae), there are eighteen *Rhodnius* species described and some are difficult to identify. The aim of this article is to contribute to the specific identification of fourteen *Rhodnius* spp. through morphological characters of the external female genitalia.

**Methods:**

Female abdomens were cut transversely. The specimens were then prepared for examination by using scanning electron microscopy.

**Results:**

The careful examination of the dorsal, posterior and ventral sides revealed characteristics that allowed the identification of each of the fourteen species.

**Conclusion:**

The use of external female genitalia as characteristics are proposed as a tool for specifically identifying *Rhodnius* species, and an identification key for these species is presented.

## Background

The Triatominae subfamily is a fundamental link in the epidemiological chain of the protozoan *Trypanosoma cruzi*, which is a registered parasite for 24 families and 150 mammalian species as well as humans [1]. Among the 18 genera comprised in the Triatominae subfamily [2,3], *Rhodnius* has been the most difficult to specifically identify according to studies by Neiva and Pinto [4]; Lent *et al.*[5], Bérenger and Sigwalt [6]. *Rhodnius* species can infect by *T. rangeli* too [7].

The first two species described for the genus *Rhodnius* were *R. nasutus* and *R. prolixus*, which were described in 1859 [8]; between that year and 1979, 10 more species were added to this genus [9]. In 2003, 4 species were added to this genus [2] by rehabilitation of *R. amazonicus*[6], description of *R. stali*[5], *R. colombiensis*[10], and *R. milesi*[11]. The seventeenth and eighteenth species described for this genus were *R. zeledoni*[12] and *R. montenegrensis*[13].

Based on the literature, identifying such vectors still relies on morphological characteristic descriptions [5,10], even though genotype studies have improved significantly and now contribute to phylogenetic evaluations [14-16].

External female genitalia from the Triatominae subfamily have rarely been used to characterize triatomines [6,17] compared with male genitalia, which have been frequently used as one of the main taxonomic characteristics [5,12,18]. After studying external female genitalia from *Panstrongylus herreri*, *P. megistus*, *R. colombiensis*, *R. prolixus*, *Triatoma infestans* and *T. vitticeps* through scanning electron microscopy (SEM), Rosa *et al.*[19] validated this morphology for taxonomy.

Among species of *Rhodnius* such as *R. brethesi* and *R. pictipes* there is no difficultly in identification [9]. However, other species such, *R. nasutus, R. neglectus, R. prolixus* and *R. robustus*, are widely known as a hard task for a precise discrimination [20,21]. The present study has shown that it is possible to distinguish the four species previously referred to by using characteristics of their female genitalia.

Regarding the epidemiological importance of *Rodhnius* species from Brazil, Gurgel-Gonçalves *et al.*[22] mentioned that *R. nasutus* and *R. neglectus*, for example, may overlap in geographic distribution in the northeastern Brazil. Then the external female genitalia can help specific distinction of *Rhodnius* spp that occupy the same area.

Given such observations, external female genitalia from 14 *Rhodnius* species were studied herein. This article also offers a key designed to contribute to the taxonomy of the group, and may later work for phylogenetic studies on this subfamily and genus.

## Methods

*Rhodnius brethesi* (N = 2), *R. colombiensis* (N = 5), *R. domesticus* (N = 7), *R. ecuadoriensis* (N = 2), *R. milesi* (N = 3), *R. montenegrensis* (N = 7), *R. nasutus* (N = 4 ), *R. neglectus* (N = 25) *R. neivai* (N = 2), *R. pallescens* (N = 2), *R. pictipes* (N = 3), *R. prolixus* (N = 15), *R. robustus* (N = 7) and *R. stali* (N = 3) were examined by SEM for this study.

These specimens were deposited or maintained in colonies at the Insetário de Triatominae da Faculdade de Ciências Farmacêuticas, Universidade Estadual Paulista (UNESP) - Araraquara and Laboratório Nacional e Internacional de Referência em Taxonomia de Triatomíneos/FIOCRUZ/Rio de Janeiro. They were killed and cut transversely at the beginning of the abdomen. They were then washed, dehydrated using an alcohol-based compound, and ovendried at 50°C. Next, they were fixed on aluminum supports at the fifth abdominal segment such that the posterior portion was at a 90-degree angle with the support base. Next, metal sputtering was used for 80 seconds at 10 mA. Thereafter, the dorsal, ventral and posterior sides were examined using a Topcon SM 300 scanning electron microscope, Topcon corporation, Hasunuma-cho, Tokyo Itabashi-ku, Japan. The comparative study was performed using images from 87 samples.

Origins for the samples are shown in Tables 1 and 2 summarizes the different characteristics between the 14 *Rhodnius* species*.* All features herein described as differential characteristics were checked for at least 15 (except for *R. ecuadoriensis* (2), *R. milesi* (3) and *R. neivai* (2)) insects per species by light microscopy (OM) to evaluate if there was intraspecific variability. Characteristics with interspecific variability were discarded from the description.

**Table 1:** Species, colony and origin of the triatomines used for the characterization of female genitalia by scanning electron microscopy

**Species**	**Colony**	**Origin**	**Initiated**
*R. brethesi*	222	Igarapé Tucunaré, Rio Curiduri, Barcelos,	20/07/2009
		AM, Brazil	
*R. colombiensis*	050	Tolima, Colômbia	15/02/2001
*R. domesticus*		Instituto René Rachou, Belo Horizonte, MG, Brazil	
*R. ecuadoriensis*		Laboratório Nacional e Internacional de Referência em Taxonomia de Triatomíneos, RJ, Brazil	
*R. milesi*		Belém, PA, Brazil	
*R. montenegrensis*	088	Montenegro, RO, Brazil	29/09/2008
*R. nasutus*	053	Patú, Messias Targino e Almino Afonso, RN, Brazil	23/05/1983
*R. neglectus*		Laboratório Nacional e Internacional de Referência em Taxonomia de Triatomíneos, RJ, Brazil	
*R. neivai*		Laboratório Nacional e Internacional de Referência em Taxonomia de Triatomíneos, RJ, Brazil	
*R. pallescens*	070	Barro Colorado, Panamá	14/12/1984
*R. pictipes*	071	Jacundá, PA, Brazil	23/05/1983
*R. prolixus*	074	Venezuela	25/05/1983
*R. prolixus*	075	Instituto Nacional de Salud, Bogotá, Colômbia	15/12/1976
*R. prolixus*	079	Ortiz Caseiro, Edoguarica, Venezuela	05/09/1999
*R. robustus*		Laboratório Nacional e Internacional de Referência em Taxonomia de Triatomíneos, RJ, Brazil	
*R. robustus*	083	Instituto de Medicina Tropical - Peru	30/08/1973
*R. stali*		Laboratório Nacional e Internacional de Referência em Taxonomia de Triatomíneos, RJ, Brazil	

**Table 2:** **Main characteristics found in the dorsal, posterior, and ventral views of the external female genitalia of 14 *Rhodnius *** **species**

**Species**	**Dorsal view**	**Posterior view**	**Ventral view**
*R. domesticus*	Curved line between 7th and 8th segments (Figure 1A)	Moon-shaped line between the 9th and 10th segments (Figure 2A)	9th segment ends slightly below the tenth segment (Figure 3A).
*R. ecuadoriensis*	Curved line between 7th and 8th segments (Figure 1B)	No transverse 1 + 1 slits on the line between 9th and 10th segments (Figure 2B)	Points of 8th gonocoxite projected onto the 9th segment (Figure 3B)
*R. milesi*	Curved line between 7th and 8th segments (Figure 1C)	Transverse 1 + 1 slits on the line between 9th and 10th segments (Figure 2C)	9th and 10th segments finish at the same plane (Figure 3C).
*R. nasutus*	Curved line between 7th and 8th segments (Figure 1D)	Transverse 1 + 1 slits on the line between the 9th and 10th segments (Figure 2D)	Semi-circular 10th segment (Figure 3D)
*R. neivai*	Curved line between 7th and 8th segments (Figure 1E)	10th segment separated by a slit in 1 + 1 lobes (Figure 2E).	7th segment line with V-shaped depression; 10th segment with slit (Figure 3E).
*R. pictipes*	Curved line between 7th and 8th segments (Figure 1F)	Rectangular 10th with a slit between 9th and 10th segments (Figure 2F)	9th segment with wide 1 + 1 lateral edges (Figure 3F)
*R. prolixus*	Curved line between 7th and 8th segments (Figure 1G)	Circular line between 9th and 10th segments (Figure 2G).	Curved 1 + 1 posterior edges of the 9th segment (Figure 3G).
*R. stali*	Curved line between 7th and 8th segments (Figure 1H)	Rectangular 10th segment with no slit in the middle (Figure 2H)	9th segment with narrow 1 + 1 lateral edges (Figure 3H)
*R. montenegrensis*	Straight line between 7th and 8th with 1 + 1 triangular points, and a trapezoidal shaped 8th segment (Figure 1I)	Short 9th segment (Figure 2I)	Circular line between 7th segment and 8th gonocoxites (Figure 3I)
*R. robustus*	Straight line between 7th and 8th with small triangular points and rectangular shaped 8th segment (Figure 1J).	Long 9th segment (Figure 2J)	Line between 7th segment and the 8th gonocoxites curved in middle (Figure 3J)
*R. brethesi*	Line between 7th and 8th segments that is slightly curved in middle (Figure 1K)	10th segment separated into 1 + 1 lobes by a cavity (Figure 2K)	8th gonocoxites are separated from 8th gonapophyses (Figure 3K)
*R. colombiensis*	Line between 7th and 8th segments that is slightly curved in middle (Figure 1L)	2 + 2 appendages along line between the 8th and 9th segments (Figure 2L)	8th gonocoxites are not separated from 8th gonapophyses (Figure 3L).
*R. neglectus*	Line between 7th and 8th segments that is slightly curved in middle (Figure 1M)	Line between 9th and 10th segments that is oval shaped in the anterior portion and which widens on the sides (Figure 2M)	Line of 7th segment is curved on the sides, but not in the middle (Figure 3M).
*R. pallescens*	Line between 7th and 8th segments that is slightly curved in middle (Figure 1N)	Oval shaped line between the 9th and 10th segments (Figure 2N)	Line of 7th segment has 1 + 1 curves on the sides and is elevated in the middle (Figure 3N)

## Results

The dorsal, posterior, and ventral sides of the 14 *Rhodnius* species were analyzed using SEM.

Evaluating the images for the *Rhodnius* species’ dorsal sides aided in defining three primary characteristics:

1) The line that divides the seventh and eighth segments;

2) The shape of the ninth segment; and

3) The shape of the tenth segment.

After visualization of the posterior side *Rhodnius* species showed four primary characteristics:

1) The line that divides the eighth and ninth segments;

2) The line that divides the ninth and tenth segments;

3) The shape of the ninth segment; and

4) The shape of the tenth segment.

Examining the ventral side showed five primary characteristics:

1) The line that divides the seventh segment and the eighth gonocoxites and gonapophyses;

2) The shape of the eighth gonocoxites;

3) The shape of the eighth gonapophyses;

4) The shape of the ninth segment; and

5) The shape of the tenth segment.

**Figure 1:**
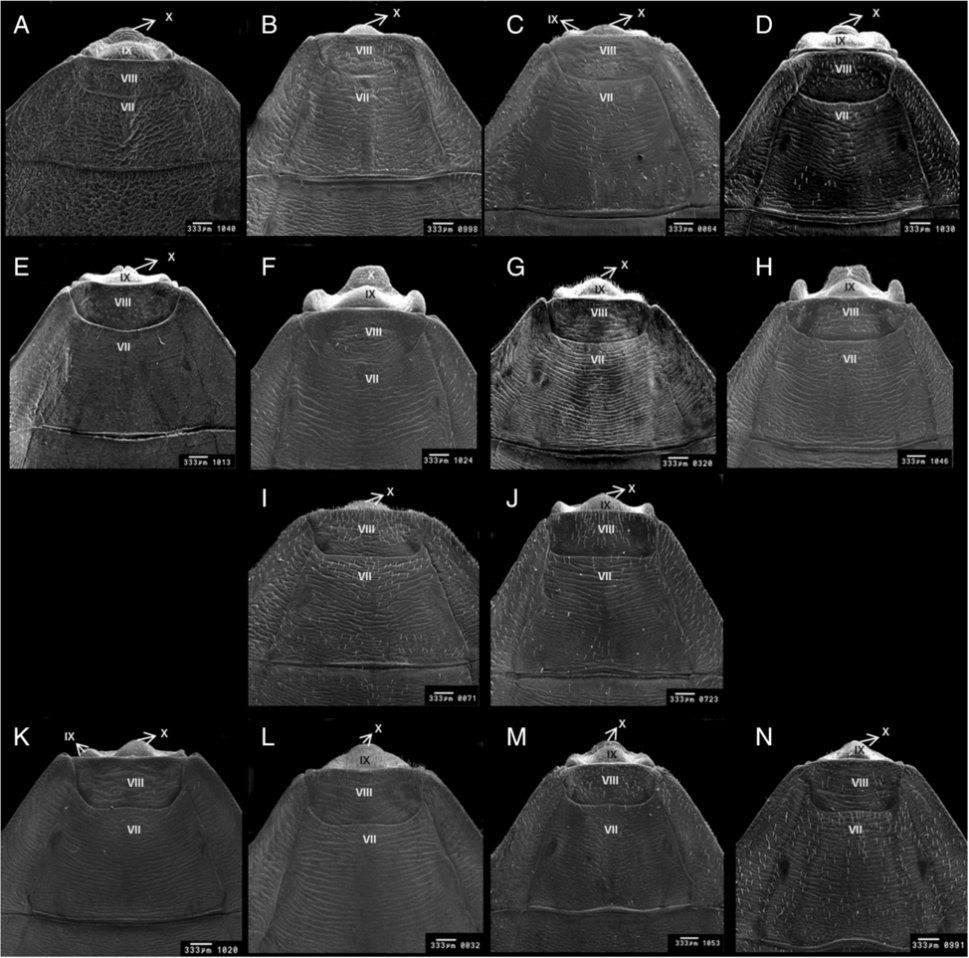
**Female external genitalia of fourteen species of *Rhodnius *by scanning electron microscopy, dorsal side. A**: *Rhodnius domesticus*; **B**: *Rhodnius ecuadoriensis*; **C**: *Rhodnius milesi*; **D**: *Rhodnius nasutus*; **E**: *Rhodnius neivai*; **F**: *Rhodnius pictipes*; **G**: *Rhodnius prolixus*; **H**: *Rhodnius stali*; **I**: *Rhodnius montenegrensis*; **J**: *Rhodnius robustus*; **K**: *Rhodnius brethesi*; **L**: *Rhodnius colombiensis*; **M**: *Rhodnius neglectus* and **N**: *Rhodnius pallescens*; VII, VIII, tergites; IX and X segments.

Based on the characteristics presented by the line that divides the seventh and eighth segments in the dorsal perspective, the species were categorized into three groups:

a) Species with a curved dividing line: *R. domesticus*, *R. ecuadoriensis*, *R. milesi*, *R. nasutus*, *R. neivai*, *R. pictipes*, *R. prolixus* and *R. stali* (Figure 1A-H);

b) Species with a straight dividing line between the seventh and eighth segments: *R. montenegrensis* and *R. robustus* (Figure 1I,J); and

c) Species with a curved dividing line and an additional downward curve in the middle: *R. brethesi*, *R. colombiensis*, *R. neglectus* and *R. pallescens* (Figure 1K-N).

Based on these criteria, a key was developed to identify the 14 *Rhodnius* species, which are described below.

**Figure 2:**
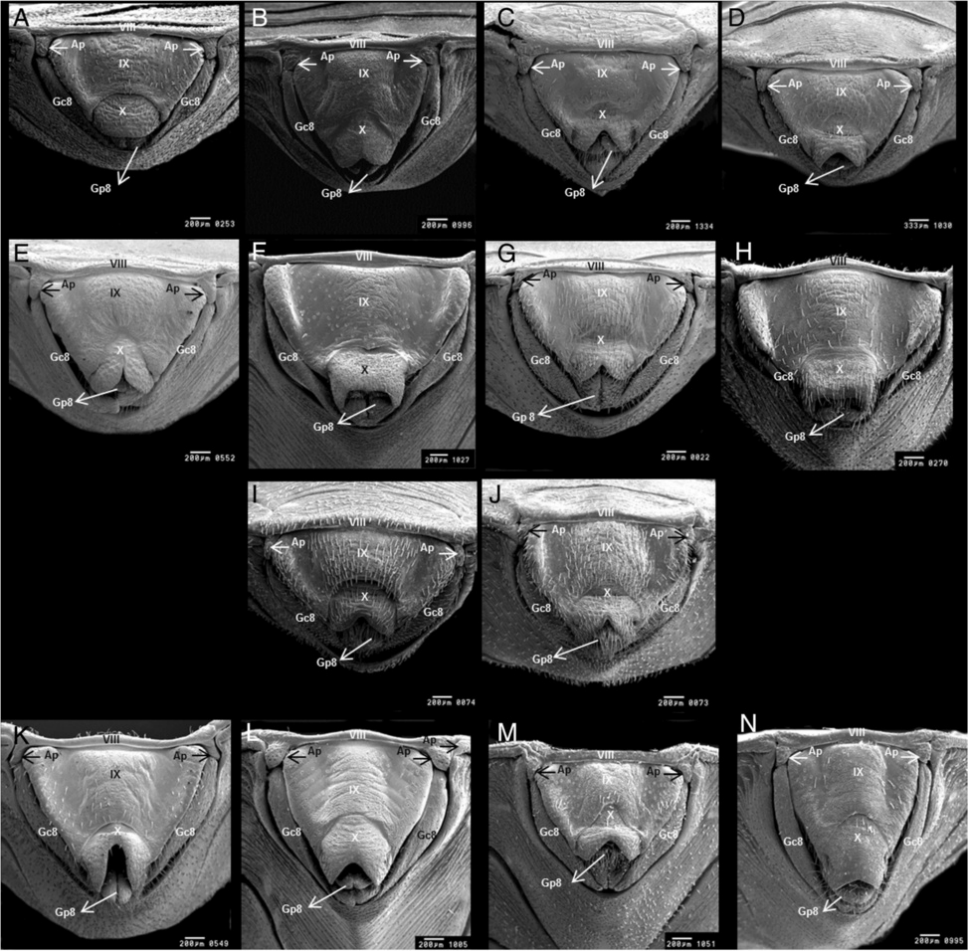
**Female external genitalia of fourteen species of *Rhodnius *by scanning electron microscopy, posterior side. A**: *Rhodnius domesticus*; **B**: *Rhodnius ecuadoriensis*; **C**: *Rhodnius milesi*; **D**: *Rhodnius nasutus*; **E**: *Rhodnius neivai*; **F**: *Rhodnius pictipes*; **G**: *Rhodnius prolixus*; **H**: *Rhodnius stali*; **I**: *Rhodnius montenegrensis*; **J**: *Rhodnius robustus*; **K**: *Rhodnius brethesi*; **L**: *Rhodnius colombiensis*; **M**: *Rhodnius neglectus* and **N**: *Rhodnius pallescens;* Ap: appendice*;* Gc8: gonocoxite 8; Gc9: gonocoxite 9; Gp8: gonapophyse 8; Gp9 gonapophyse 9; VIII tergite; IX and X segments.

**Figure 3:**
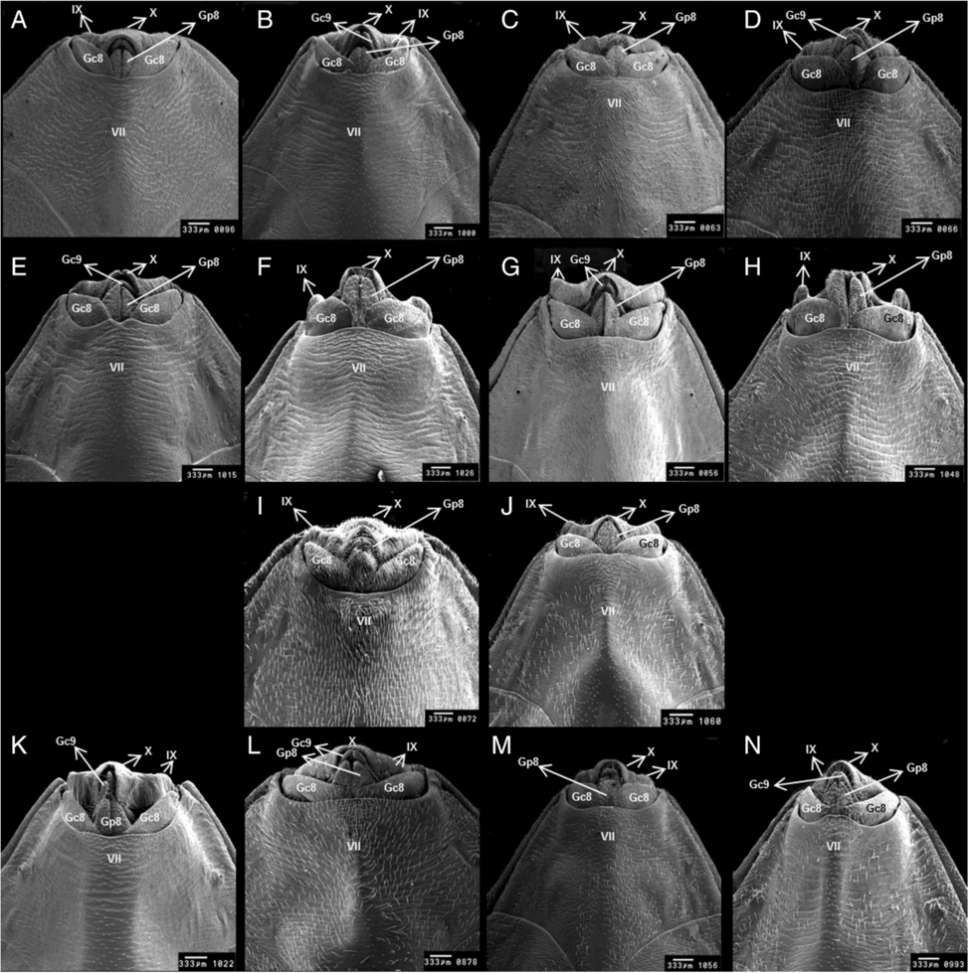
**Female external genitalia of fourteen species of *Rhodnius *by scanning electron microscopy, ventral side. A**: *Rhodnius domesticus*; **B**: *Rhodnius ecuadoriensis*; **C**: *Rhodnius milesi*; **D**: *Rhodnius nasutus*; **E**: *Rhodnius neivai*; **F**: *Rhodnius pictipes*; **G**: *Rhodnius prolixus*; **H**: *Rhodnius stali*; **I**: *Rhodnius montenegrensis*; **J**: *Rhodnius robustus*; **K**: *Rhodnius brethesi*; **L**: *Rhodnius colombiensis*; **M**: *Rhodnius neglectus* and **N**: *Rhodnius pallescens;* Gc8: gonocoxite 8; Gc9: gonocoxite 9; Gp8: gonapophyse 8; Gp9 gonapophyse 9; VII sternite; IX and X segments.

I) The eight species in group ***a*** were separated by traits observed on the posterior and ventral sides; the results are as follows. The posterior perspective showed that *R. domesticus* has a moon-shaped line that divides the ninth and tenth segments (Figure 2A). The ventral perspective showed that the ninth segment ends slightly below the tenth segment (Figure 3A).

*Rhodnius milesi* has lateral transverse 1 + 1 slits on the line that divides the ninth and tenth segments (Figure 2C). The ventral perspective showed that the ninth and tenth segments (Figure 3C) end on the same plane.

*R. ecuadoriensis* does not include slits on the tenth posterior segment (Figure 2B). The ventral perspective showed that eighth gonocoxites ends projected to the ninth segment (Figure 3B).

The tenth segment of *R. neivai* is clearly separated into two lobes by a central slit in the posterior portion (Figure 2E). The ventral perspective showed that the eighth gonocoxites and gonapophyses have a V-shaped depression in the middle, and the tenth segment includes a slit in the middle (Figure 3E).

*Rhodnius nasutus* has 1 + 1 lateral slits along the posterior end of the tenth segment (Figure 2D). The ventral perspective showed that the tenth segment is semi- circular in shape (Figure 3D).

The posterior perspective showed that the tenth segments in *Rhodnius pictipes* and *R. stali* are rectangular. In *R. pictipes*, a slit was observed at the dividing line between the ninth and tenth segments (Figure 2F). *R. stali* did not include this slit (Figure 2H). The ventral perspective showed that *R. pictipes* includes a ninth segment with wide lateral edges (Figure 1F, 3F), while in *R. stali*, these edges are narrow (Figure 1H, 3H).

The posterior perspective for *Rhodnius prolixus* showed that the line dividing the ninth and tenth segments is circular (Figure 2G). On the ventral side, the posterior 1 + 1 edges for the ninth segment are curved (Figure 3G).

II) The two species that compose group ***b*** were separated by traits observed in the dorsal, posterior and ventral portion.

The dorsal perspective showed that the seventh segment for *R. montenegrensis* forms 1 + 1 lateral triangular points along the edge of the eighth segment, and the eighth segment is trapezoid-shaped (Figure 1I). In *R. robustus*, the triangular points are smaller, and the eighth segment is rectangular (Figure 1J). From a posterior perspective, it is easier to distinguish between the ninth segment, which is longer in *R. robustus* than *R. montenegrensis* (Figure 2I,J). The ventral perspective showed that the dividing line between the seventh segment and eighth gonocoxites is circular in *R. montenegrensis* and curved in the middle of *R. robustus* (Figure 3I,J).

III) The four species in group ***c*** were distinguished by traits on the posterior and ventral sides.

The posterior perspective showed that *R. pallescens* includes an oval dividing line between the ninth and tenth segments (Figure 2N). In *R. neglectus*, this line is also oval-shaped at the beginning, but it widens on the posterior sides (Figure 2M). The ventral perspective showed that *R. pallescens* has a dividing line between the seventh segment and eighth gonocoxites with 1 + 1 lateral curves and a pronounced middle elevation (Figure 3N). *Rhodnius neglectus* includes the same lateral curves but not the middle elevation (Figure 3M).

The posterior perspective showed that the tenth segment of *Rhodnius brethesi* is separated into 1 + 1 lobes by a cavity (Figure 2K), while *R. colombiensis* has 2 + 2 appendages along the dividing line between the eighth and ninth segments (Figure 2L). The ventral perspective showed that *R. brethesi* includes eight gonocoxites that are separate from the eighth gonapophyses (Figure 3K). This separation was not observed in *R. colombiensis* (Figure 3L).

The dorsal and ventral side cuticles for the *Rhodnius* species studied had transverse linear grooves, except for the *R. domesticus* cuticle, which comprised irregular grooves (Figure 1A).

## Discussion

Even though female genitalia structures can be observed by OM, SEM has several advantages over optical. For this purpose it was possible to view the three dimensional external shape of a structure (in this case the female genitalia) in the same image. Electron microscopy also allows us to focus on many details of the structure. After checking them by OM, observations described the species specific features, and discarded the polymorphic ones.

Among the 18 *Rhodnius* species [2,12,13], the female external genitalia for *R. amazonicus*, *R. dalessandroi*, *R. paraensis* and *R. zeledoni* were not studied because specimens were unavailable.

The relevance of the male genitalia in specific identification of triatomines has beeen widely used by many authors, including Lent and Jurberg [18] to describe new species. On the other hand the female genitalia, also evaluated by other authors [6,9], has shown itself less valuable due to the hard task of the dissection technique, unlike the male genitalia. The first publication in 2010 using SEM involving non-dissected insects of non-closely related triatomine species (*P. megistus*, *P. herreri*, *R. prolixus, R. colombiensis, T. infestans* and *T. vitticeps*), clearly showed that these species can be distinguished by this features [19]. However, in 2012 the validity of this approach was confirmed for closely related species (*R. robustus* and *R. montenegrensis*) [13]. In an unpublished masters thesis, Simone Caldas Neves used the same approach to distinguish a recently described species (*T. jatai*[23], in the theses called T. n.sp.) closely related to *T. costalimai*[24].

Given the difficulties for specific distinctions in the *Rhodnius* species [4,9,12] this study was performed to increase the number of morphological traits that can be used to identify the species in this genus.

For this study, details previously published for external female genitalia traits in four species were reconsidered; the species included were *R. colombiensis*, *R. prolixus*[19], *R. montenegrensis* and *R. robustus*[13].

The key presented and summarized in Table 2 was developed using the most evident traits to identify the 14 *Rhodnius* species using the external female genitalia. Three groups of species (***a***, ***b***, and ***c***) were formed according to characteristics of the dividing line between the seventh and eighth dorsal segments, which is a visible and perceptible feature (Figure 1A-N). However, given the information verified by the 42 figures, the 14 species can be identified using traits on the dorsal, posterior, and ventral sides as either isolated or associated characteristics.

In the key, *R. montenegrensis* and *R. robustus* separation is based on the dorsal, posterior, and ventral sides (Figures 1I,J, 2I,J, 3I, and J). Though eight species from group ***a*** and four species from group ***c*** were characterized based on their posterior and ventral sides, it is important to note that these species also include characteristics on the dorsal side, as with *R. nasutus* and *R. neivai*, which have distinct tenth segment shapes (Figure 1D,E).

Thus, the dorsal side shows that the eighth, ninth and tenth segments are also distinct among the 14 species evaluated (Figure 1A-N).

Based on a posterior perspective, the 14 species can be distinguished by the dimension and shape of the eighth gonocoxites and gonapophyses, the ninth and tenth segments as well as the dividing lines between the eighth and ninth as well as the ninth and tenth segments. This perspective shows that the posterior portion of the tenth segment is concave at the end in 11 species (Figure 2B-E,G,I-N); in *R. pictipes* and *R. stali*, this segment is straight at the end (Figure 2F,H), while in *R. domesticus*, it is semi- circular (Figure 2A).

A ventral perspective shows that the 14 species have distinctive lines at the end of the seventh segment as well as shapes and dimensions for the gonapophyses, the gonocoxites, as well as the ninth and tenth segments. From this perspective, 10 species have a dividing line between the seventh segment and eighth gonocoxites as well as gonapophyses, which is curved at the sides and convex in the middle (Figure 3C-H,J,L-N). In the remaining four species, this line is curved (Figure 3A,B,J, and K). In six species, the eighth gonocoxites meet in a triangular shape (Figure 3A,B,G, and I-K); in the other eight species, this point is non-triangular (Figure 3C-F, H, and L-N). The eighth gonapophyses are triangular in 12 species (Figure 3A,E,G, and I-N) and rod-shaped in two species (Figure 3F,H).

After combining the results herein on the external female genitalia for these 14 *Rhodnius* species using the five complexes established by Carcavallo *et al*. [20] for this genus, the following factors can be considered.

A comparative analysis of the external female genitalia characteristics for the *R. dalessandroi* complex was impossible because only *R. milesi* specimens were examined.

*Rhodnius pictipes* and *R. stali*, which compose the *R. pictipes* complex, include traits that join and exclude similar species. These traits include a dividing line between the seventh and eighth segments from the dorsal perspective, rectangular shape of the tenth segment from the posterior perspective, as well as ninth and tenth segment features (Figures 1F,H, 2F, and H). From the ventral perspective, similarities were observed for the line that divides the seventh segment from the eighth gonocoxites and gonapophyses as well as the shape of the eighth gonocoxites and tenth segments (Figures 1F,H, 2F,H, 3F, and H). However, each of these two species maintained distinguishing characteristics. From the dorsal perspective at the intersection between the lines that separate the seventh segment and connectives, *R. pictipes* include 1 + 1 triangles that are not evident in *R. stali*. The dorsal perspective also shows that *R. stali* comprise salient and straight lateral 1 + 1 edges on the ninth segment, while in *R. pictipes*, these edges are wide (Figure 1F,H). From the ventral perspective, this difference is clarified by the shape of the ninth segment end portion, which is wide in *R. pictipes* and straight in *R. stali* (Figure 3F,H.)

For the *R. prolixus* complex, which comprises *R. prolixus*, *R. domesticus*, *R. nasutus*, *R. neglectus*, *R. robustus*, and, more recently, *R. montenegrensis*, the analyses confirmed that the connecting traits include the shape of the ventral line that divides the seventh segment from the eighth gonocoxites and gonapophyses, except for *R. montenegrensis*. The species include distinguishing traits on the three sides studied. A distinct trait was verified for *R. domesticus*, which was the only species of the 14 that does not include transversely grooved cuticles (Figure 1A). Importantly, *R. neglectus* and *R. prolixus*, which are particularly difficult to distinguish, do have distinguishing characteristics on the dorsal side; the line that separates the seventh and eighth segments is completely curved in *R. prolixus*, while in *R. neglectus*, it is curved on the sides and lightly convex in the middle (Figure 1G,M). On the posterior side, the difference between these two species evident through the dividing line between the ninth and tenth segments, which is circular in *R. prolixus* and oval at the beginning in *R. neglectus* but widens on the posterior sides (Figure 2G,M). The ventral side of *R. prolixus* shows a laterally expanded ninth segment, which was not observed in *R. neglectus* (Figures 3G,M).

*Rhodnius colombiensis*, *R. ecuadoriensis* and *R. pallescens*, which comprise the *R. pallescens* complex, include a set of ninth and tenth segments on the posterior side that form an isosceles triangle (Figure 2B,L, and N). This configuration clearly connects these three species. The distinction between such species is particularly evident on the ventral side, where the posterior portion of the eighth gonocoxites and gonapophyses is projected on the ninth segment in *R. ecuadoriensis*; however, the gonocoxite is not projected in *R. colombiensis*. This structure has a different shape in the two species (Figure 3B,L). In *R. pallescens*, the dividing line that separates the seventh segment from the eighth gonocoxites and gonapophyses is elevated in the middle; the elongated shape of the ninth and tenth segments also distinguishes this species from *R. colombiensis* and *R. ecuadoriensis* (Figure 3N,L, and B).

The studies performed herein facilitate identification and descriptions for three distinguishing features on the dorsal side, four features on the posterior side and five features on the ventral side of the external female genitalia in the 14 *Rhodnius* species, which distinguish the species. Using such findings, a key was developed to aid in distinguishing the 14 *Rhodnius* species*.* There is no record in the literature for intraspecific polymorphism for the female genitalia; however, this characteristic has not been widely explored. Given that, we do not discard the possibility of intraspecific variation for field populations and some of the features elected shall be confirmed with a much larger sample of field material. Therefore, we consider the possibility of further adjustments in the key. On the other hand, all descriptive studies in the morphology field also have this limitation. For triatomines, morphological variations, not described before, were detected for *Rhodnius nasutus* in Ceara [25], and *Triatoma rubrovaria* in Rio Grande do Sul [26].

The morphological traits for the external female genitalia validate four of the five complexes proposed by Carcavallo *et al*. [20] for the genus *Rhodnius*. They also specifically distinguish the species in the complexes. The fifth complex proposed by Carcavallo *et al.*[20], which also includes the species *R. milesi* was impossible to evaluate due to a lack of *R. dalessandroi* specimens.

It is important to highlight that the results herein corroborate the observations in Rosa *et al*. [19], which considered these traits for *P. herreri*, *P. megistus*, *R. colombiensis*, *R. prolixus*, *T. infestans* and *T. vitticeps* and validated the taxonomy. However, the study herein is a continuation of the study on the posterior ventral abdominal segments for the female nymphs in the fifth nymphal stage from six Triatominae species in Rosa *et al.*[27], wherein specifically distinguishing characteristics were identified.

Finally, it will necessary to clarify whether the features of the female genitalia described by SEM may also be observed through OM by non-experts. In addition, given the possible polymorphism for field populations we recommend: (i) first using to the traditional classification and then (ii) make use of this study for the specific confirmation.

## Conclusions

Examination by SEM of the dorsal, posterior and ventral surfaces of the female external genitalia of 14 species of *Rhodnius* enabled the identification.

Through OM was also possible to identify the 14 species of *Rhodnius*.

## Competing interests

The authors declare that they have no competing interest.

## Authors’ contributions

Conceived the study: JAR. Selected the bugs: VJM, SG, DBC, JO, JDN and HP. Prepared samples: VJM, SG, DBC, JO, JDN and HP. Analysed data: JAR, CG and JMSB. Interpreted data: JAR, VJM, SG, DBC, JO, JDN, HP, MCP, MC, CG and JMSB. Wrote the manuscript: JAR, VJM, SG, DBC, JO, JDN, HP, MCP, MC, CG and JMSB. All authors read and approved the final version of the manuscript.
